# A Case of Dislocation of the Os Femoris into the Ischiatic Notch, in a Female

**Published:** 1844-01-13

**Authors:** Wm. Keith

**Affiliations:** Philadelphia


### A CASE OF DISLOCATION OF THE OS FEMORIS

*Into the Ischiatic Notch, in a Female.*

BY WM. KEITH, M. D.

To the Editor of the Medical Examiner.

Not having had my attention particularly directed to the subject, I was somewhat surprised at the facts stated, in the last Examiner, in relation to the "*infrequency of dislocations of the os femoris in the female*;" and as I can furnish an instance of the kind, to add to the very small number on record, perhaps you will not deem me obtrusive in making the following brief statement.

While practising in the West not long since, I met with a case of luxation of the femur into the ischiatic notch. The patient was a female, in the fourth month of her pregnancy.

I had the satisfaction of reducing the dislocation in the presence of Drs. Mitchell, Ward, Morehead, and Bell, of Zanesville, Ohio.

Ten days after the accident, the lady was able to walk without the use of crutches, and in due season was happily delivered of a promising child.

*Philadelphia*, December 26th, 1843.

